# Hip and spine bone mineral density are greater in master sprinters, but not endurance runners compared with non-athletic controls

**DOI:** 10.1007/s11657-018-0486-9

**Published:** 2018-07-03

**Authors:** J. Piasecki, J. S. McPhee, K. Hannam, K. C. Deere, A. Elhakeem, M. Piasecki, H. Degens, J. H. Tobias, A. Ireland

**Affiliations:** 1 Sport, Health and Performance Enhancement Research Centre, Nottingham Trent University, Nottingham, UK; 2 Department for Sports and Exercise Sciences, Manchester Metropolitan University, John Dalton Building, Chester Street, Manchester, M1 5GD UK; 3 Musculoskeletal Research Unit, School of Clinical Sciences, University of Bristol, Bristol, UK; 4 MRC/ARUK Centre of Excellence for Musculoskeletal Ageing Research Centre, School of Medicine, University of Nottingham, Derby, UK; 5 Institute of Sport Science and Innovations, Lithuanian Sports University, Lithuania, Lithuania

**Keywords:** Exercise, Mechanoadaptation, Athlete, Physical activity

## Abstract

**Summary:**

We examined bone density in older athletes and controls. Sprinters had greater hip and spine bone density than endurance athletes and controls, whereas values were similar in the latter two groups. These results could not be explained by differences in impact, muscle size or power between sprint and endurance athletes.

**Purpose:**

We examined the relationship between prolonged participation in regular sprint or endurance running and skeletal health at key clinical sites in older age, and the factors responsible for any associations which we observed.

**Methods:**

We recruited 38 master sprint runners (28 males, 10 females, mean age 71 ± 7 years), 149 master endurance runners (111 males, 38 females, mean age 70 ± 6 years) and 59 non-athletic controls (29 males, 30 females, mean age 74 ± 5 years). Dual X-ray absorptiometry was used to assess hip and spine bone mineral density (BMD), body composition (lean and fat mass), whilst jump power was assessed with jumping mechanography. In athletes, vertical impacts were recorded over 7 days from a waist-worn accelerometer, and details of starting age, age-graded performance and training hours were recorded.

**Results:**

In ANOVA models adjusted for sex, age, height, body composition, and jump power, sprinter hip BMD was 10 and 14% greater than that of endurance runners and controls respectively. Sprinter spine BMD was also greater than that of both endurance runners and controls. There were no differences in hip or spine BMD between endurance runners and controls. Stepwise regression showed only discipline (sprint/endurance), sex, and age as predictors of athlete spine BMD, whilst these variables and starting age were predictive of hip BMD.

**Conclusions:**

Regular running is associated with greater BMD at the fracture-prone hip and spine sites in master sprinters but not endurance runners. These benefits cannot be explained by indicators of mechanical loading measured in this study including vertical impacts, body composition or muscular output.

## Introduction

The bone adapts to the mechanical loading it experiences during every day physical activity (PA) and exercise, with higher impacts associated with intense PA being advantageous for bone strength [1–3]. Older people are usually less active than young and what activities they do engage with tend to be low impact and therefore of little benefit to bone [4]. This age-related decline in physical activity likely contributes to declining bone strength. Indeed, positive associations have been reported between PA levels and bone strength in older adults [4, 5], suggesting that exercise is an effective way to improve and maintain bone mineral density (BMD) and bone strength in older individuals. However, several interventions designed to improve bone strength through exercise training have failed to show clinically significant effects [6]. A possible explanation for this is that bone adaptation in adults is slow and effects of exercise may take several years to fully manifest [7]. There is also uncertainty over the types of activities that are potentially osteogenic.

Master athletes offer a model to examine associations between long-term exercise training and bone strength, and have the added advantage that comparison can be made between different disciplines to determine which activities are more osteogenic. In young adults, the benefits of regular exercise have been suggested to depend upon the type of activity, being greater in high impact activities such as sprinting whereas little benefit is evident in lower impact activities such as walking, cycling, or swimming [8–10]. This may also be the case for older adults. For example, master cyclists have a higher incidence of osteopenia and lower hip and spine BMD than non-athletic controls [11]. In contrast, male and female sprinters had 15 and 18% greater trabecular BMD in the distal tibia than non-athletes [12], whereas benefits in male and female endurance runners were 7 and 9%, respectively. It remains unclear whether the benefits of sprint and endurance running are also observed in older age for the hip or lumbar spine, fractures of which represent a major disease burden. In a small study of master athletes, total body, arm, trunk pelvis, legs, thoracic, and lumbar spine regional BMD were greater in sprint athletes than controls with no advantages evident in endurance runners [13]. Previous studies have omitted comparisons with controls, considered younger athletes and been limited by small sample size or not investigated these regions [12–14].

To the extent that observed associations between discipline and BMD reflect a response to exercise, different benefits of distinct running events on BMD are likely to be related to differences in skeletal loading by muscle and reaction forces between those activities. For example, the larger reaction [15, 16] and muscle forces in sprinting could explain the greater benefits to bone in sprint compared to endurance running. Direct assessment of vertical impacts and indirect indicators of muscular loading (lean mass and muscle power) in sprint and endurance runners would provide relevant information to test this hypothesis.

It was hypothesised that both athletic groups have greater bone strength than controls, with the largest advantages in sprinters. In addition, to the extent that any observed differences were a consequence of exercise participation, it was expected that the larger bone advantages in sprint than in endurance athletes are attributable to differences in physical activity (accelerometry data) and muscle mass and function. To investigate this, we compared hip and spine BMD between master sprinters, master endurance runners and non-athletic controls. We also examined differences in the number of vertical impacts and indicators of mechanical loading such as body composition and muscle power, and through ANCOVA and multiple linear regression models examined to what extent these could explain group differences.

## Materials and methods

### Study design

Master athletes (MA) were recruited at nationwide athletics competitions as part of a multiple cohort study named “VIBE” and included male and female athletes aged ≥ 60 years currently competing in sprint, middle or long distance running and in the 12 months preceding recruitment had competed at regional level or higher. Regional ethics approval (14/NW0275) was obtained prior to the study and written informed consent was obtained from all participants.

MAs were classified as sprinters (28 male and 10 female) if competing in events less than 800 m in distance, or endurance athletes (111 male and 38 female) if competing in events greater than or equal to 800 m in distance. Each athlete completed a questionnaire to determine demographics, lifestyle, their past physical activity behaviours, physical activity at the time of wearing the accelerometer. The questionnaire data allowed us to group athletes according to years trained consecutively: (1) those training all of their life through childhood, (2) those training since 18 years old, (3) those training since 30 years old and (4) those training since 50 years old. Mean age-graded performance (AGP) was determined by taking the athlete’s highest ranked performance within the last 2 years, and expressing it as a percentage of the world record for that age and distance. AGP ranged from 77 to 92% across the cohort, indicating a high level of performance relative to respective age group records. For example, a marathon of 3 h and 30 min at the age of 70 gives an age-graded performance of 80%.

The MAs were drawn as a sub-sample from a larger study that included 286 MAs with accelerometry measurements and of those, 189 participants also additionally completed DXA assessments at the Manchester research centre. These 189 participants with both accelerometry and DXA data were included in the present study. The DXA images from two participants were excluded due to movement artefacts, so data are presented from 187 individuals with valid DXA and accelerometry data.

Control participants were individuals recruited as part of the EU “MYOAGE” study [17] using advertisements in newspapers and University of the Third Age with the aim to recruit socially active individuals. Volunteers were excluded if: dependent living, unable to walk a distance of 250 m, presence of morbidity (such as neurologic disorders, metabolic diseases, rheumatic diseases, heart failure, severe chronic obstructive pulmonary disease and haemocoagulative syndromes), immobilisation for 1 week during the last 3 months, orthopaedic surgery during the last 2 years and/or suffering from pain or functional limitations.

### DXA scans

Standing height was measured to the nearest millimetre and body mass was measured to the nearest 0.1 kg. Whole body, total hip and lumbar spine dual energy X-ray absorptiometry (DXA) scans were performed using a DXA scanner while the participant lay supine (Lunar Prodigy Advanced, GE Healthcare, encore version 10.50.086). During the measurements, a light cotton t-shirt was worn by the participants to reduce measurement errors due to clothing absorption. Body composition (fat mass and lean mass) was measured from total body scans, whilst bone mineral density (BMD, g cm^−2^) was measured from hip and spine scans. All measurements were recorded after manual adjustment of the regions of interest carried out offline. Repeat total body and hip DXA scans were performed in eight MAs within 1 month of the original scan. Using these repeat scans the short-term error for our laboratory was 2.0% for hip BMD and 0.9% for spine BMD.

### Muscle function

A Leonardo Mechanography Ground Reaction Force Platform (Leonardo Software version 4.2: Novotiec Medical GmbH, Pforzheim, Germany) was used to assess lower limb muscle function during a vertical jump as described previously [18]. From this, we were able to assess both absolute and relative power. Briefly, the participants performed a two-footed countermovement jump where each participant was asked to jump as high as they could. Jumps were performed with a trained assistant present and in reach of the participants in case of a fall or falter. Each participant repeated the jump sequence three times, with approximately 30 s rest between jumps. The jump with the maximum power was used for statistical analysis.

### Accelerometry

Accelerometry data was collected from the athletes only. Each athlete received a GCDC × 16–1c (Gulf Coast Data Concepts, Waveland, Mississippi) which was placed in a Velcro strap and worn around the waist with the accelerometer device placed over their right hip. Each athlete wore this monitor for 7 consecutive days, only removing it when showering, bathing, swimming and sleeping. The monitor was kept on for all other daily activities including athletic training. Time sheets were completed over the 7-day period to identify the time the monitor was first worn, the time it was removed in the evening and to indicate any reason why that day was not of their usual routine. Accelerometers were configured with standardised settings prior to participant use with a sampling frequency of 50 Hz, a deadband setting of 0.1 g (the threshold which must be exceeded before a recording is made) and a timeout setting of 10 s (meaning that a single sample every 10 s is taken even if the recording is < 0.1 g) [19]. Once the period of use was completed, the participant returned the accelerometer to the centre, by post, where the raw accelerometry data was then uploaded to a secure shared drive and read into Stata 13 (StataCorp, College Station, TX). A standardised cleaning and processing procedure was used and is described in detail elsewhere [19]. In short, the *Y*-axis accelerations data were cleaned to remove movement artefacts and any periods of nil data collection, presumably due to the participant not wearing the accelerometer. Activity data were normalised based on 7 valid days of 14 h with ≥ 10-h recording time. *Y*-axis peaks were calculated based on accelerations higher than the previous and subsequent reading and recorded within 14 pre-specified *g* bands. These were condensed to three impact bands; low (≥ 0.5 to < 1.0 g), medium (≥ 1.0 to < 1.5 g) and higher (≥ 1.5 g) impact. All *g* values represent *g* over and above 1 g from earth’s gravitational force [4].

### Statistical analysis

Statistical analysis was performed using SPSS for Windows (v21, IBM, USA). Data was firstly assessed for normality of distribution using P-P and Q-Q graphs, and the Kolmogorov-Smirnov test. Accelerometry data was not normally distributed, so this data was log transformed for further analysis. Non-normally distributed data are presented as median (25th/75th) quartiles and all other data are presented as mean ± standard deviation (SD).

Univariate ANOVA analysis with Fisher’s Least Significant Difference post-hoc tests was used to identify differences between the three groups (sprinters, endurance runners and controls). Males and females were combined in the statistical analysis and differences were determined with adjustment for sex. There was no evidence of group *sex interactions (*P* > 0.7 in all cases); therefore, data from both sexes were combined for analysis. Differences were considered significant at *P* < 0.05. Lean mass [20] and muscle function [21] are highly correlated with bone strength, even when accounting for allometric scaling. Therefore, these and other co-variates were included to assess group differences in bone outcomes using a series of five different models, as shown in Table 3. Model 1: age, height, sex; model 2: model 1 + fat mass; model 3: model 1 + lean mass; model 4: model 1 + lean mass + fat mass; model 5: model 4 + absolute power.

To further investigate factors associated with bone outcomes in the athletes, single factor linear regression was performed for each individual variable (age, height, AGP, training age, hours trained, fat mass, lean mass, body mass, absolute power, vertical impacts (low, medium and high), discipline and sex) in relation to hip and spine BMD, for the athlete groups combined. Next, a stepwise linear regression was conducted with the athlete groups combined, using the same variables, to determine predictors of hip and spine BMD within Master Athletes. Results of regression analyses are presented as standardised regression coefficients (*ß*) and 95% confidence interval unless otherwise stated.

## Results

### Participant characterisation

Participant characteristics are shown in Table 1. Controls were older than both sprint and endurance runners. There was no difference between any groups in height. Endurance runners were lighter and had lower BMI than both sprinters and controls, and sprinters also had lower BMI than controls. Controls had 32 and 40% higher body fat percentage than sprinters and endurance runners, respectively. Sprinters had greater lean mass and 10–30% greater relative and absolute power values than both endurance runners and controls. Lean mass but not be absolute or relative power was also greater in endurance runners than controls.

**Table 1:** Participant characteristics separated by group and sex. Values are mean ± standard deviation; *P* values for post hoc comparisons between groups are shown after adjusting for sex

Variable	Group						Group pair-wise comparisons		
	1. Sprint		2. Endurance		3. Controls		1 vs 2	1 vs 3	2 vs 3
Sex	M	F	M	F	M	F			
*N*	28	10	111	38	29	30			
Age (years)	70.9 ± 6.4	71.5 ± 7.9	69.9 ± 5.7	69.1 ± 50	74.1 ± 5.7	73.3 ± 4.5	0.181	*0.022*	*< 0.0005*
Height (cm)	174 ± 6	162 ± 6	173 ± 6	162 ± 7	172 ± 9	160 ± 5	0.467	0.104	0.180
Body mass (kg)	74.3 ± 9.7	62.9 ± 10.9	67.9 ± 7.7	56.0 ± 7.4	80.2 ± 16.2	63.1 ± 11.5	*< 0.0005*	0.098	*< 0.0005*
BMI (kg m^−2^)	24.5 ± 2.7	23.9 ± 3.8	22.5 ± 3.1	21.4 ± 2.1	27.1 ± 4.7	24.5 ± 4.2	*< 0.0005*	*0.012*	*< 0.0005*
Lean mass (kg)	57.8 ± 5.6	43.8 ± 4.7	54.2 ± 5.4	41.3 ± 5.0	52.2 ± 8.4	38.1 ± 4.3	*0.001*	*< 0.0005*	*0.007*
Fat (%)	17.3 ± 5.7	25.0 ± 10.3	15.6 ± 5.7	22.5 ± 7.2	29.9 ± 8.7	34.2 ± 9.0	0.136	*< 0.0005*	*< 0.0005*
Absolute power (kW)	2.64 ± 0.90	1.68 ± 0.59	2.03 ± 0.56	1.41 ± 0.38	2.19 ± 0.59	1.46 ± 0.49	*< 0.0005*	*0.002*	0.179
Relative power (W kg^−1^)	35.3 ± 10.4	27.3 ± 11.0	30.1 ± 8.1	25.2 ± 6.0	27.5 ± 5.2	23.0 ± 4.9	*0.002*	*< 0.0005*	0.060

Italics represent significance at *p* < 0.05

### Characteristics related to athletic training

Mean age-graded performance ranged was 82.2% across the athlete cohort, indicating a high level of performance as shown in Table 2. Age-graded performance was greater in sprinters than in endurance runners. There was no difference in the number of hours per week trained between sprinters and endurance. The number of impacts recorded in the low and medium bands were 2.2- and 3.0-fold higher, respectively, in endurance than sprint athletes, but the number of counts in band 3 (high impacts) did not differ between endurance and sprinters.

**Table 2:** Athlete-specific characteristics, separated by athletic group and sex. Values are mean ± standard deviation except accelerometry counts (median (IQR)); *P* values for post hoc comparison between groups are shown after adjusting for sex

Variable		Group				Group difference *P*
		1. Sprint		2. Endurance		
Sex		M	F	M	F	
Training age (years)	< 18 18–29 30–49 > 50	17 2 6 3	5 1 1 3	57 12 22 18	8 3 11 15	0.140
Current training hours per week	0–1 1–3 4–7 7+	0 5 18 4	0 2 4 3	2 13 59 36	0 4 19 15	0.111
Age-graded performance (%)		82.3 ± 13.6	89.5 ± 11.4	76.5 ± 10.7	80.6 ± 10.2	*0.002*
Accelerometry low impact (0.5–1 g) counts		20,876 (12362–40,738)	14,368 (6623–33,408)	40,882 (28228–53,412)	37,161 (25787–55,780)	*<0.0005*
Accelerometry medium impact (1–3.5 g) counts		6434 (2364–13,692)	3326 (694–13,081)	33,458 (18847–49,909)	29,868 (21076–41,859)	*< 0.0005*
Accelerometry (counts) high impact (> 3.5 g) counts		131 (9–693)	37 (4–293)	193 (20–1038)	90 (12–774)	0.291

Italics represent significance at *p* < 0.05

### Bone mineral density

In minimally adjusted model 1, mean hip BMD in sprinters was ~ 10% greater than endurance runners and 9% greater than controls (Table 3). Adjustment for fat mass in models 2, 4 and 5 increased the differences between sprinters and controls, whilst adjustment for lean mass in model 3 had little effect on group differences. There were no differences in hip BMD between endurance and controls for any model (all *P* > 0.15).

**Table 3:** Bone outcomes separated by group and sex. Values are mean ± standard deviation; *P* values for post hoc comparison between groups are shown after adjustment for sex. Adjustments: M1, adjusted for sex, height, and age; M2, M1 + fat mass; M3, M1 + lean mass; M4, M1+ fat mass + lean mass; M5, M4+ absolute power

Variable	Group							Group pair-wise comparison		
	1. Sprint		2. Endurance		3. Controls		Model	1 vs 2	1 vs 3	2 vs 3
	M	F	M	F	M	F				
Hip BMD (g cm^−2^)	1.15 ± 0.16	0.97 ± 0.11	1.03 ± 0.15	0.88 ± 0.11	1.05 ± 0.12	0.88 ± 0.13	1 2 3 4 5	< 0.0005 < 0.0005 < 0.0005 < 0.0005 0.001	0.006 0.001 0.016 0.002 0.007	0.184 0.953 0.159 0.993 0.980
Spine BMD (g cm^−2^)	1.21 ± 0.21	1.02 ± 0.18	1.09 ± 0.13	0.89 ± 0.03	1.15 ± 0.17	0.95 ± 0.15	1 2 3 4 5	< 0.0005 < 0.0005 0.002 0.001 0.004	0.110 0.001 0.345 0.009 0.018	0.010 0.763 0.005 0.957 0.923

**Fig. 1:**
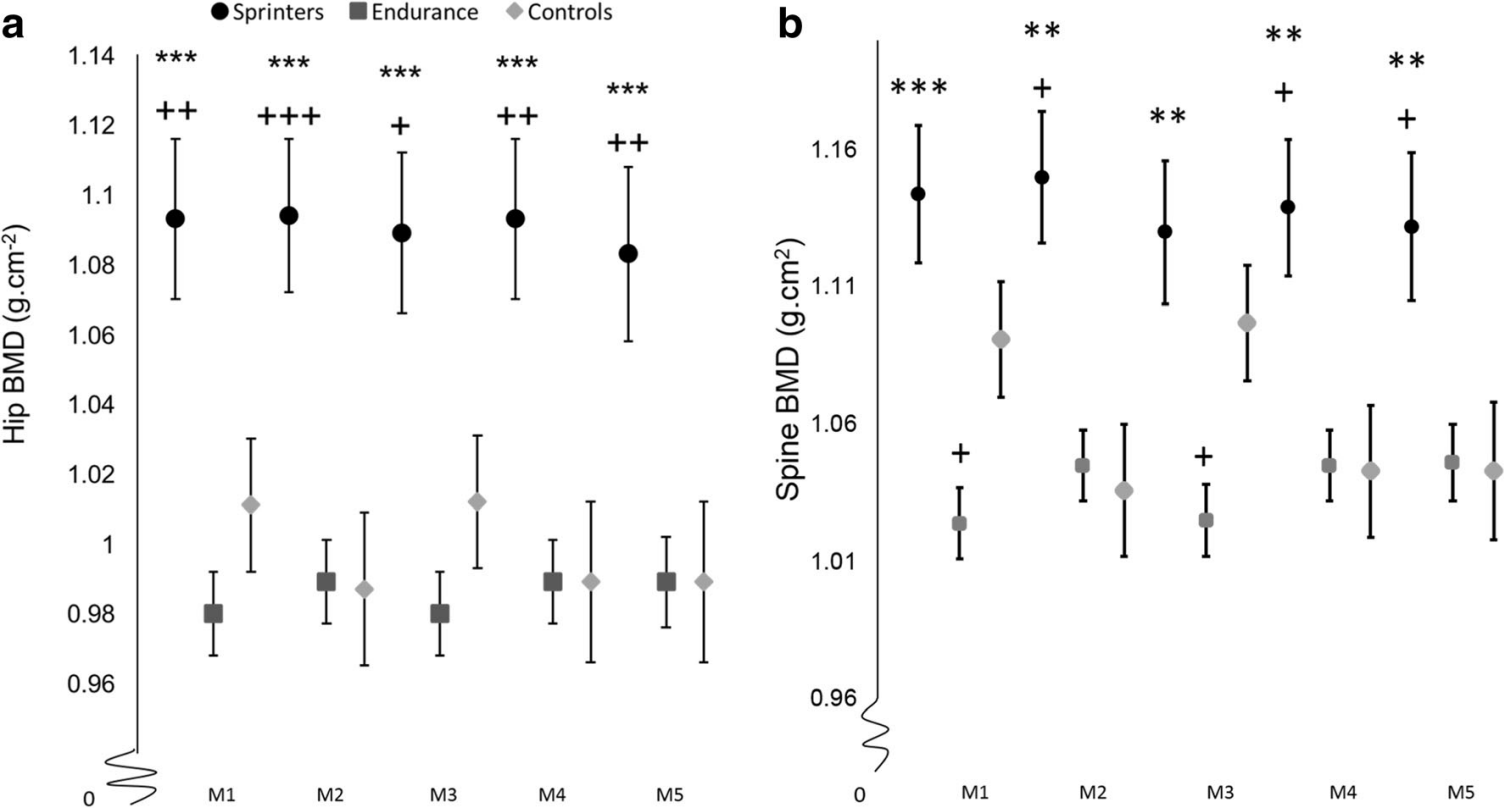
Adjusted mean estimates separated by group in a series of ANOVA models (means ± SD) for **a** the hip and **b** the spine BMD adjustments: M1; adjusted for sex, height, and age, M2; M1 + fat mass, M3; M1 + lean mass, M4; M1+ fat mass + lean mass, M5; M4+ absolute power. Asterisks indicate significant difference from endurance *P < 0.05, *P < 0.01, ***P < 0.001. Crosses indicate significant difference from controls +*P* < 0.05, ++*P* < 0.01, +++*P* < 0.001

Sprinters had greater spine BMD than endurance athletes did in model 1 and this remained the case after further adjustment in models 2, 3, 4 and 5. There was no difference in spine BMD between sprinters and controls in minimally adjusted model 1 or after lean mass adjustment in model 3. However, adjustment for fat mass in models 2, 4 and 5 showed values to be higher in sprinters than controls. Conversely, greater spine BMD was found in controls than endurance runners in models 1 and 3, but these group differences were fully attenuated by adjustment for fat mass in models 2, 4 and 5. The adjusted means for each model of adjustment are presented in Fig. 1a, b.

### Regression analysis

Results of linear regressions between individual athlete characteristics and bone outcomes, when adjusted for age, height, body mass and sex are shown in Table 4. Discipline (sprinter), AGP and absolute jump power were positively associated with hip BMD, whilst later starting age, low and medium impact counts were negatively associated with hip BMD. Discipline (sprinter), training age, and fat mass were positively associated with spine BMD, whilst a later starting age, low and medium impacts were negatively associated with spine BMD.

**Table 4:** Results of linear regression between each individual athlete characteristic and bone outcomes in athletes only, adjusting for age, height, and sex presented as standardised regression coefficient (*β*)

Variable		Hip BMD				Spine BMD			
		*β*	95% CI		*P*	*β*	95% CI		*P*
Discipline (sprinter)		0.281	0.155	0.408	0.000	0.268	0.145	0.390	0.000
AGP		0.131	− 0.004	0.266	0.059	0.100	− 0.029	0.229	0.130
Training age^1^		− 0.231	− 0.366	− 0.096	0.001	− 0.153	− 0.284	− 0.021	0.024
Training hours		0.017	− 0.118	0.151	0.809	0.017	− 0.109	0.144	0.791
Fat mass		0.102	− 0.032	0.235	0.137	0.230	0.104	0.356	< 0.001
Lean mass		0.093	− 0.168	0.354	0.485	0.216	− 0.039	0.471	0.099
Absolute jump power		0.150	− 0.005	0.304	0.060	0.174	0.023	0.326	0.025
Accelerometry counts	Low	− 0.177	− 0.040	− 0.314	0.012	− 0.246	− 0.117	− 0.375	0.000
	Medium	− 0.169	− 0.032	− 0.306	0.016	− 0.296	− 0.169	− 0.422	0.000
	High	0.109	− 0.034	0.252	0.137	− 0.056	0.080	− 0.193	0.420

^1^Test for linear trend between categories

In stepwise multiple linear regressions, the variables identified as predictors of hip BMD were sex (greater values in males, standardised regression coefficient 0.393, 95%CI 0.257 to 0.529, *P* < 0.001), discipline (greater values in sprinters, 0.246, 95%CI 0.113 to 0.38, *P* < 0.001), age (− 0.259, 95% CI, − 0.128 to − 0.39, *P* < 0.001) and starting age (− 0.168, 95%CI − 0.03 to − 0.307, *P* = 0.012). For spine BMD sex (male, 0.527, 95%CI 0.4 to 0.654, *P* < 0.001), discipline (sprinter, 0.248, 95%CI 0.121 to 0.374, *P* < 0.001) and age (− 0.13, 95% CI − 0.003 to − 0.257, *P* = 0.046) were identified as predictors. Similar results were obtained when all variables were entered simultaneously (results not shown).

## Sensitivity analyses

To examine the influence of regional lean mass on bone, we also performed analyses adjusted for appendicular or lower limb lean mass rather than whole body measures. In addition, we performed analyses with lean and fat mass indices (lean or fat/height^2^ respectively), BMI and relative jump power. Results of these alternative analyses (data not shown) were similar to those described above; therefore, whole body measures and unadjusted body composition and peak power values were retained in analyses.

## Discussion

The main finding was that hip and spine BMD were greater in sprinters than endurance athletes and non-athletic controls. These differences remained after adjustments for body composition and muscle function. Endurance athletes had lower spine BMD than controls during initial analysis, but this difference disappeared after adjusting for body fat. These findings suggest that long-term endurance exercise has little benefit for hip and spine BMD. In contrast, long-term sprint training was associated with greater hip and spine BMD than non-athletic controls. This is the first study to compare hip and spine BMD of older master athletes from different training disciplines and controls in a large cohort. The hip and spine are important clinically because they are prone to fracture in old age. Previous studies were limited by the absence of a control group [14] or discipline-specific comparisons [10, 22], recruitment of middle-aged athletes [13] or focussed on distal or less fracture-prone regions rather than hip and spine [12, 13, 22].

Our findings support previous observations of greater BMD in sprinters compared with endurance runners and controls [13, 14] [12]. A previous DXA study in younger master athletes (40–64 years) reported similar bone outcomes for endurance athletes and controls, whilst distal tibia trabecular BMD as assessed by pQCT was greater for both sprint and endurance runners compared to controls, highlighting regional adaptions of the tibia [12]. It could be that the effect of endurance running on BMD diminishes with older age, as identified in other sports [23]. However, studies of elite young adult endurance runners have also found no benefit to hip and spine BMD [24]. The differences between hip and tibia adaptations to different forms of running could be explained by the biomechanics of running at different speeds. Knee and hip torques increase with increasing running speed, but the torque around the ankle tends to plateau at speeds above 5 m per second [25].

With regard to the discipline-specific advantages in hip BMD of sprinters which we and others observed [13, 14, 26], sprinters had higher lean mass and jumping power than endurance runners and controls. Though absolute and relative jumping power were positively associated with both hip and spine BMD, this relationship was no longer observed once discipline was included in the regression, which other than sex and age was the only independent predictor of BMD at both sites. Taken together, these observations suggest that differences in muscle function likely contribute to observed BMD differences between sprinters and endurance runners, but its influence is only partially explained by muscle power as measured by jumping mechanography. This limitation may reflect that whilst we measured a number of parameters relevant to bone loading that were previously shown to be associated with bone outcomes [4, 7, 21, 27, 28], we were not able to directly assess bone deformation, nor the loads placed upon bones by reaction and muscle forces. A previous study employing detailed biomechanical assessment of running gait in sprint athletes identified kinetic variables as predictors of bone strength within a master sprinter population [29]. More detailed biomechanical analyses within different athletic populations may identify relevant components of the training stimulus. Moreover, BMD is influenced by lifelong exposure to mechanical strain, as indicated by greater hip and spine BMD in retired youth athletes [30] at old age. Our muscle measures were only obtained at a single point in time relatively late in life. Given the known decrease in muscle bulk and function with age [17] particularly in athletes [23], our study may have significantly underestimated differences in muscle function between these two groups across the life-course.

Mechanical loading on the skeleton is a reflection not only of muscle function, but also of participation in physical activity. High impact activities, even of relatively few occurrences each day, are thought to be osteogenic based on positive associations found between high vertical impact activity and bone outcomes in non-athletic older individuals [5, 31]. Our expectation was that sprinters would achieve greater numbers of high impacts than endurance athletes which was hypothesised to contribute towards their greater BMD. Whereas BMD was substantially higher in sprinters, the endurance athletes and sprinters had similar numbers of high impacts as measured using accelerometry. It should be noted that the accelerometers only registered vertical impacts and not horizontal components of acceleration. Indeed, the power output and, most likely, the magnitude and rate of strains experienced by the bones during sprinting, are greater than those during endurance running predominately due to the horizontal rather than vertical impulses [32]. Further research is needed to test whether overall (horizontal and vertical) accelerations are associated with bone adaptations observed in sprint but not endurance runners.

It is also conceivable that vertical impacts of lower magnitude, in the low and medium range, exert osteogenic activity. We previously reported higher levels of low and medium vertical impact activity in master athletes compared to controls [33], and in the present study endurance runners showed even greater numbers of low and medium impacts compared to sprinters. However, BMD in endurance runners was similar to that of controls and below that of sprinters. Indeed, low and medium impacts were inversely related to BMD. This inverse relationship may reflect our recent observation that low and medium impacts as recorded here are inversely related to BMI [34], of which the latter is positively related to bone mass [35]. Our observation that spinal BMD was in fact lower than controls in minimally adjusted models, which differences attenuated after adjustment for fat mass, is consistent with this explanation. The absence of bone benefits in endurance runners could also be related to desensitisation of the bone by regular low-level habitual activity [36], and/or saturation of the response to high-magnitude loading after a very small number of loading cycles [37, 38]. Therefore, the higher levels of low and medium-impact activity performed by endurance than sprint and control athletes may not contribute positively to bone strength.

An alternative explanation to mechanical influences explaining the difference between sprint and endurance athletes’ BMD could be a pre-existing self-selection bias in sport participation, possibly relating to aspects of body stature not captured by our methodology but otherwise influencing BMD. This possibility has been proposed in a number of previous master athlete studies [10, 12, 39], but never explored. Studies of bone health in individuals beginning to take part in sprint and endurance events either in childhood or in adulthood could examine whether such bias exists.

### Strength and limitations

The main strength of this paper is the comparison of a large cohort of elite level master athletes competing at very high levels and with extensive training history of different disciplines, and controls. This allowed us to assess the impact of muscle strength, body mass, body composition and vertical impacts on the BMD at the hip and spine, sites which are clinically important due to their susceptibility to bone fractures in old age. Previous studies have omitted comparisons with controls, considered younger athletes or did not investigate these fracture-prone regions [12–14]. However, the cross-sectional nature of the study limits assessment of causal relationships between type of sport and BMD due to possible uncontrolled confounders. For instance, we had little information about other factors potentially related to bone health, such as use of medications and nutrient intake including vitamin D, but it seems unlikely that these will have differed substantially between groups so as to explain the BMD differences we observed. Similarly, differences in hormones such as testosterone may have influenced bone health, particularly in endurance athletes with low body fat. These factors were not measured, although limited evidence suggests that testosterone and growth factor levels are higher in master athletes of similar age and body composition [40]. In addition, a detailed training log was not taken, so we may have missed some additional information about differences in exposure to higher impacts between sprinters and endurance runners. Another consideration is displacement of the accelerometer during training in extreme high impacts, affecting accuracy of readings.

### Conclusions

Master sprint runners have greater BMD at the fracture-prone hip and spine sites, and greater lean mass and muscle power than healthy non-athletic controls, but no such advantages in BMD were evident in endurance runners. BMD advantages in sprinters were only partly explained by differences in lean mass and muscle function, whilst further adjustment for other indicators of skeletal loading including accelerometry measures within sprinters and endurance runners could not explain group differences. Further studies are required to identify to what extent discipline-specific advantages in BMD relate to pre-existing differences in skeletal health, or to variance in skeletal loading not captured in this study.
